# Vitrectomy surgery of diabetic retinopathy complications

**Published:** 2016

**Authors:** Brănişteanu Daniel Constantin, Bilha Andrei, Moraru Andreea

**Affiliations:** * Ophthalmology Department, “Gr. T. Popa” University of Medicine and Pharmacy, Iasi, Romania,; ** ”RETINA CENTER” Eye Clinic, Iasi, Romania,; *** Ophthalmology Clinic, “N. Oblu” Clinical Emergency Hospital, Iasi, Romania

**Keywords:** diabetic retinopathy, 20/23G pars plana vitrectomy, vitreous hemorrhage, retinal detachment

## Abstract

Purpose: To assess the anatomical and functional results after vitreoretinal surgery, in a large number of patients with complications due to diabetic retinopathy. Also, to compare the 23G vs. the 20G surgical procedures in these cases, regarding efficacy, facility, safety, and postoperative recovery.

Methods: Interventional, retrospective, comparative study of cases operated for different complications of diabetic retinopathy between January 2000 and December 2014. All cases were operated under a local anesthesia by the same surgeon, by using standard 20G Vitrectomy (between January 2000 and October 2011) and ambulatory 23G vitrectomy (since November 2011). Cases had a complete ophthalmic evaluation and were followed- up for at least 12 months.

Results: 1.267 eyes of 1.129 patients were operated between January 2000 and December 2014. 23G vitrectomy was performed in 578 eyes. The mean age in the study group was of 57.49 ± 14.17 years (ranging from 16 to 78 years old), with a male/ female ratio of 0.916. The surgery indications were represented by media opacities (609 cases – 48.06%), vitreoretinal tractions and detachments (583 cases – 46.01%), persistent macular edema (38 cases – 3%) and persistent neovascularization with rubeosis (37 cases – 2.93%). A final anatomical success was obtained in 1174 cases (92.65%). Preoperative best corrected visual acuity (BCVA) (less or equal to counting fingers in 936 eyes - 73.87%), improved postoperatively in 923 eyes (72.84%), stabilized in 201 eyes (15.86%), and decreased in 143 eyes (11.28%). At a final examination, 932 eyes (73.55%) had a BCVA equal or better to 0.1. Cases operated with the 23G vitrectomy had a shorter surgery and a quicker postoperative recovery. Overall, simpler cases like vitreous hemorrhage and epimacular membranes had a better anatomical and functional result as compared to long standing or macular involvement detachments. The main intra and postoperative complications, lower with the 23G vitrectomy, were represented by iatrogenic retinal breaks, recurrent hemorrhages, redetachment, and neovascular glaucoma.

Conclusions: These results confirmed the efficacy and safety of vitreoretinal surgery in improving most complications of diabetic retinopathy on a large series. With modern, less invasive techniques, the chance of a better surgery and also a quicker patient recovery increased significantly.

## Introduction

Diabetic retinopathy is one of the leading causes of visual loss in both the elderly and the working-age population. Danaei et al. recently reported in “The Lancet” that age-standardized adult diabetes prevalence has reached 9.8% in men and 9.2% in women [**1**]. Approximately 24% of these patients are already diagnosed with different forms of diabetic retinopathy but 28% will remain undiagnosed until the onset of complications [**2**,**3**].

The prevalence of diabetic retinopathy grows proportionally to the duration of diabetes, so all the patients with type 1 diabetes and 60% of those with type 2 diabetes will be diagnosed with a form of diabetic retinopathy after 20 years of disease [**4**].

The diabetic retinopathy affects the retinal microvascularization, leading to progressive retinal ischemia, neovascularization and fibro- cellular proliferation. Many patients are referred to a retina specialist in late phases of diabetic retinopathy evolution, when severe complications like vitreous hemorrhage and tractional retinal detachment are already installed. On the other hand, 5% of the patients with diabetic retinopathy, appropriate ophthalmic care, and strict metabolic control still develop ocular complications requiring a surgical treatment.

The first pars plana vitrectomy was successfully performed in 1970, on a diabetic eye with persistent vitreous hemorrhage, by Robert Machemer, and led to a significant increase of the anatomical and functional prognosis in these cases. This outstanding evolution towards ophthalmic microsurgery [**5**] led to surgical instruments miniaturization and the refinement of surgical techniques. Today, minimally invasive small G transconjunctival pars plana vitrectomy (with either 23G, 25G or 27G) is the standard of care in such cases. All this time, non-clearing vitreous hemorrhage remained one of the main indications of vitrectomy in diabetic eye. Today, the advances in surgical techniques allowed the improvement of most complex cases of retinal detachments. The other indications for surgery, such as persistent neovascularization and refractory macular edema have faded in time as intravitreal therapy with anti-VEGF agents and steroids proved to be more efficient, easier, and safer.

The purpose of this paper was to assess the anatomical and functional results after vitreoretinal surgery in a large series of patients operated for complications due to diabetic retinopathy, and to compare the 23G versus 20G surgical procedure regarding efficacy, facility, safety, and postoperative rehabilitation.

## Material and methods

The present study was interventional, retrospective, and comparative. The patients were included if one of the following complications due to diabetic retinopathy was present: non- clearing vitreous hemorrhage, vitreomacular traction syndrome (epiretinal membranes, retinal detachments, and macular heterotopia), persistent neovascularization with rubeosis iridis, persistent or tractional macular edema.

All the patients were operated between January 2000 and December 2014. Between January 2000 and October 2011, the standard 20G vitrectomy was performed by using the Accurus/ Alcon equipment at the Ophthalmology Department in “St. Spiridon” Hospital, Iasi. Between November 2011 and December 2014, the procedure was performed exclusively on ambulatory basis, by using 23G vitrectomy provided by Constellation/ Alcon unit in “Retina Center” private practice, Iasi. All the cases were operated under local anesthesia by the same surgeon (B.D.C.). The sub-Tenon’s anesthesia was mainly used during the 20G vitrectomy, and peribulbar anesthesia was performed to complete the 23G vitrectomy.

The anticoagulants and antiplatelet medication was stopped, reduced, or temporarily replaced in the perioperative period.

Cases were clinically followed-up for at least 12 months. At each visit, a complete ophthalmic evaluation was performed by including the best-corrected visual acuity (BCVA), intraocular pressure and according to each case, ultrasonography, or spectral domain optical coherence tomography (SD-OCT).

The complexity of the surgery varied largely according to the severity of each case, and included complete gestures such as vitreous removal, membrane peeling (with or without dye enhancement)segmentationand/ordelamination of neovascular pegs, endodiathermy, endolaserphotocoagulation, subretinal fluid removal and air, gas or silicone oil endotamponade.

## Results

The study involved 1267 eyes of 1129 patients who were operated for different complications of diabetic retinopathy during 15 years’ experience. According to the authors’ knowledge, this was the largest series described in Romanian literature. Among these patients, 540 were men and 589 were women, the ratio between the two being statistically insignificant (0.916).

The mean age of patients was 57.49 years ± 14.17 years (with limits between 16 and 78 years old). The majority of patients, 864 (76.52%), had type 2 diabetes.

**Fig. 1:**
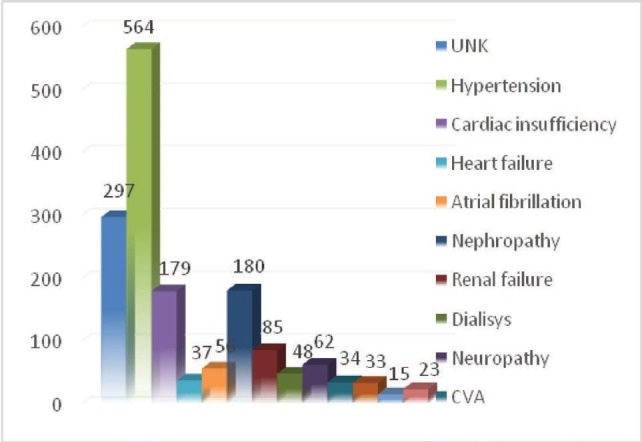
Associated systemic conditions

From 1129 patients included in this study, 832 (73.69%) had one or more associated systemic conditions (**Fig. 1**). The most frequent associated conditions were arterial hypertension (49.95%), cardiac failure (15.85%), and diabetic nephropathy (15.94%).

**Fig. 2:**
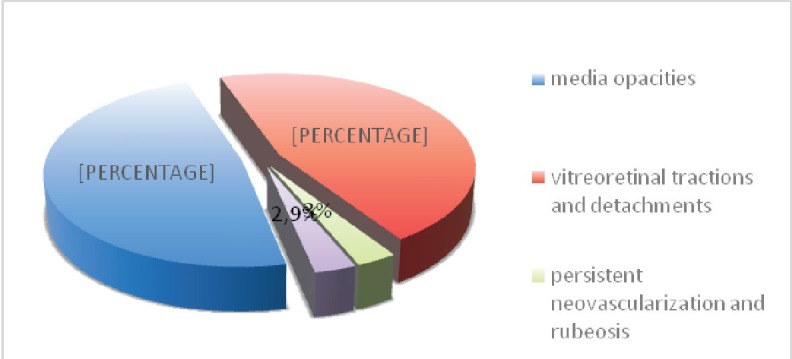
Distribution of cases according to surgical indi- cation

The main indications for surgery were vitreous opacities (609 cases – 48.06%), vitreoretinal tractions and retinal detachments (583 cases – 46.01%) (**Fig. 2**). The other indications included: persistent retinal neovascularization with rubeosis iridis (37 cases – 2.93%), and persistent macular edema (38 cases – 3%).

The standard 20G vitrectomy was performed in 689 cases (54.38%), and transconjunctival 23G was performed in 578 cases (45.61%).

**Fig. 3:**
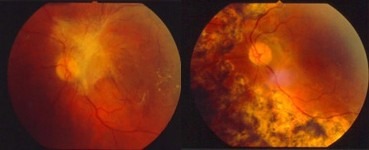
Fibrovascular membrane with macular involvement. Pre and 12 months postoperative 20G vitrectomy (2003); BCVA improved from 0.1 to 0.5

**Fig. 4:**
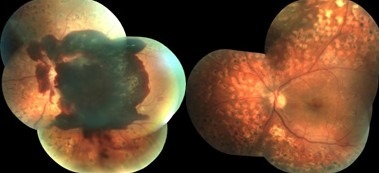
Massive preretinal hemorrhage. Pre and next day postoperative 23G vitrectomy (2013); BCVA improved from 01. to 0.5

**Fig. 5:**
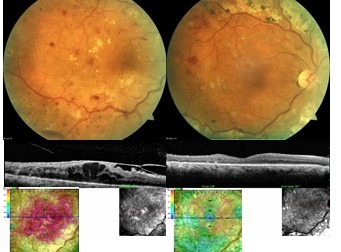
Tractional macular edema. Pre and 6 months postoperative 23G vitrectomy (2014); BCVA improved from 0.1 to 0.7

Most cases (1124 – 88.71%) had a stable anatomical result after the initial surgery (**Fig. 3**-**5**). With repeated surgical interventions, a final anatomical success was recorded in 1174 cases (92.65%). A number of 93 eyes (7.34%) were finally lost due to extensive complications.

Preoperative BCVA was less than counting fingers (0.002) in 936 cases (73.87%). Postoperatively, the BCVA improved in 923 cases (72.84%), stabilized in 201 cases (15.86%), and decreased in 143 cases (11.28%). At the last follow-up, 932 eyes (73.55%) had a BCVA of ≥ 0.1 and a mean 0.21 ± 0.16.

Cases that underwent a 23G surgery and the cases that were operated for vitreous opacities or tractions not involving the macula had a better anatomical and functional prognosis.

The main intra and postoperative complications encountered were iatrogenic breaks, cataract, recurrent hemorrhage, recurrent retinal detachment due to proliferation and neovascular glaucoma. With the conversion to the 23G technique, the number of iatrogenic breaks and recurrent retinal detachments significantly decreased. Also, the intraoperative use of anti- VEGF in selected cases significantly decreased the risks of progression to neovascular glaucoma and bleeding. Systemic complications were noticed intraoperatively in only two cases: one case of hemorrhagic stroke and one case of ischemic stroke. Only the latest, fully recovered.

## Discussions

A strict glycemic control and correction of associated conditions are mandatory to reduce the incidence of surgery in diabetic retinopathy, and also to provide better anatomical and functional results postoperatively [**6**].

The standard follow-up protocol of the diabetic patient has an important role in the early diagnosis and prevention of ocular complications. Prompt panretinal photocoagulation should be immediately performed in proliferative or severe non-proliferative diabetic retinopathy [**7**].

Pars plana vitrectomy has proved a standard of care for complications due to diabetic retinopathy cases that have been registered in the last decades. During the surgical intervention, the laser photocoagulation on the retina is completed, and, in selected cases, the intravitreal injection of anti-VEGF drugs or steroids helps reducing the angiogenesis and macular edema before, intra or postoperatively.

The development of minimally invasive vitrectomy and the integration of 23G, 25G and 27G systems into current clinical practice have led to a much efficient, quicker, and safer procedure. The transconjunctival sutureless approach has spectacularly improved the patient’s comfort and recovery. Smaller instruments and high cutting probes make small G vitrectomy highly efficient even in most complicated cases. Unlike the 20G vitrectomy probe, in small G technique, the vitrector can be used as a multifunctioning tool for cutting, segmenting, dissecting, and removing the fibrovascular membrane, as well as for aspirating blood or subretinal fluid.

Recent studies confirmed that small G vitrectomy provides better anatomical and functional results also due to reduced postoperative inflammation [**8**-**10**].

The integration of SD-OCT in our current clinical practice and the use of intravitreal anti- VEGF drugs since 2007 have also changed the approach of diabetic retinopathy complications. SD-OCT offers high resolution, cross-section images of the macula in a quick, non-invasive way. Thus, the macular thickness, the morphological structure of all layers and the vitreoretinal interface can be clearly evaluated [**11**]. We are now able to make a clear distinction between different types of macular edema and to immediately refer to surgery those cases with an obvious macular traction (**Fig. 5**).

Recent studies proved that the intravitreal administrations of anti-VEGF agents in the cases of proliferative diabetic retinopathy, persistent neovascularization and rubeosis iridis, significantly decrease neovascularization and improve macular edema [**12**]. Still, the intravitreal administration of VEGF inhibitors did not become a standard of care in proliferative diabetic retinopathy and its complications because requires frequent administration and monitoring. Anti-VEGF intravitreal administration is also associated with systemic risks. The reoccurrence of proliferation, membrane contraction, and worsening of the retinal traction are some of the reported ocular side effects.

The indications for vitrectomy in macular edema have changed in the last decade due to the use of anti-VEGF agents and SD-OCT. Still, the anti-VEGF brings no benefit in cases of tractional macular edema, in which surgery may be mandatory. Also, surgery is indicated in macular edema refractory to multiple intravitreal anti-VEGF or steroids administrations because it improves oxygen diffusion from the vitreous to the retina and decreases the quantity of intraocular VEGF.

Despite the major innovations in the diabetic retinopathy treatment, most ocular complications are still resolved by vitrectomy: non-clearing vitreous hemorrhage, tractional or combined retinal detachment, severe fibrovascular proliferations, macular heterotopia, and tractional diabetic macular edema.

The DRVS study confirmed the benefits of early vitrectomy, significantly more patients who underwent an early surgery had a better final BCVA and stable results after 4 years [**13**- **15**]. Nowadays, the proper time of surgery is individually established according to the status of the fellow eye, the degree of visual impairment, the presence of associated ocular findings, and the lifestyle of the patient.

Most frequent intraoperative complications associated with diabetic vitrectomy are iatrogenic retinal breaks and hemorrhages. The iatrogenic breaksmostlyoccurinthinoratrophicretinaduring membrane peeling, close to the tractions and have to be properly lasered all around. Hemorrhages are rare due to direct vascular injury, but more often because of neovascular peg segmentation, and are easily controlled by diathermy. The intravitreal anti-VEGF administration a few days before surgery is a useful manner to reduce the intraoperative bleeding in eyes with extensive neovascularisation.

Most frequent postoperative complications are cataract, recurrent hemorrhage (17-26%, with a higher frequency in younger patients), rubeosis iridis, and neovascular glaucoma [**16**-**22**]. The intraoperative administration of anti-VEGF drugs at the end of surgery is an easy gesture to prevent uncontrolled postoperative angiogenesis and severe complications.

The results obtained in our study, confirmed the reported literature data on a significant number of cases. A careful vitrectomy with a complete membrane removal and an intraoperative photocoagulation leads to a good anatomical and visual result in most cases. The vast majorities of cases remain stable and do not require additional surgery.

Although performed on a smaller number of patients (because of a later integration in clinical practice), the minimally invasive small G vitrectomy proved to be an excellent tool for ambulatory surgery due to its higher facility, excellent efficacy and safety, and faster recovery of the patient.

## Conclusions

In our 15 years’ experience, vitreoretinal surgery proved to be efficient and safe in improving most complications due to diabetic retinopathy. The new 23G transconjunctival vitrectomy has an enhanced feasibility as ambulatory surgery, offers a higher efficacy and comfort, and allows a faster patient recovery. Many complications of diabetic retinopathy are now medically treated, but the most severe ones still require the surgeon’s skills and state-of-the-art equipment.
